# Honokiol-induced apoptosis and autophagy in glioblastoma multiforme cells

**DOI:** 10.3892/ol.2013.1548

**Published:** 2013-08-28

**Authors:** KEN-HU CHANG, MING-DE YAN, CHIH-JUNG YAO, PEI-CHUN LIN, GI-MING LAI

**Affiliations:** 1 Division of Hematology Oncology, Department of Internal Medicine, Shuang Ho Hospital, Taipei Medical University, Taipei 116, Taiwan, R.O.C.; 2 Division of Gastroenterology, Department of Internal Medicine, Wang Fan Hospital, Taipei Medical University, Taipei 116, Taiwan, R.O.C.; 3 Center of Excellence for Cancer Research, Wan Fang Hospital, Taipei Medical University, Taipei 116, Taiwan, R.O.C.; 4 Division of Hematology and Medical Oncology, Department of Internal Medicine, Wan Fang Hospital, Taipei Medical University, Taipei 116, Taiwan, R.O.C.; 5 National Institute of Cancer Research, National Health Research Institutes, Miaoli 35053, Taiwan, R.O.C.

**Keywords:** glioblastoma multiforme, honokiol, apoptosis, autophagy

## Abstract

Honokiol, a hydroxylated biphenyl compound isolated from the Chinese herb *Magnolia officinalis*, has been reported to have anticancer activities in a variety of cancer cell lines. The present study aimed to evaluate the anticancer effect and possible molecular mechanisms of honokiol in a glioblastoma multiforme (GBM) cell line. The anticancer activities of honokiol were investigated in the DBTRG-05MG GBM cell line. The effect of honokiol on cell growth was determined using a sulforhodamine B assay. Flow cytometry and immunoblotting were used to measure honokiol-induced apoptosis (programmed cell death type I) and autophagy (programmed cell death type II). Honokiol was observed to reduce DBTRG-05MG cell viability in a dose-dependent manner. At a dose of 50 μM, honokiol markedly decreased the expression of Rb protein and led to the cleavage of poly(ADP-ribose) polymerase and Bcl-xL to promote apoptosis in the cancer cells. In addition, markers of autophagy, including Beclin-1 and LC3-II, were also significantly increased. In addition to apoptosis, honokiol was also able to induce autophagy in the DBTRG-05MG cells. The mechanisms that are responsible for the correlation between honokiol-induced apoptosis and autophagy require further investigation. Such efforts may provide a potential strategy for improving the clinical outcome of GBM treatment.

## Introduction

Glioblastoma multiforme (GBM) is the most common and invasive adult malignant brain tumor in humans. Despite standard treatments, including surgery and radiotherapy, which have been administered to patients with GBM in the past decade, the prognosis remains poor, owing to the acquired resistance of the GBM cells to apoptosis (1–3).

In the past decade, the induction of apoptosis (programmed cell death type I) has become the major strategy to combat cancer. However, resistance to apoptosis is considered to be a characteristic of several types of cancers, particularly primary GBM. Therefore, the identification of innovative strategies other than the induction of apoptosis is urgently required. Studies have demonstrated the potential of autophagy (programmed cell death type II) as a target for cancer therapy (4). The most useful chemotherapeutic agent for GBM, temozolomide, has also been reported to exert a proautophagic effect, indicating the importance of autophagy modulation in GBM treatment (5).

With the exception of traditionally synthetic compounds, numerous phytochemicals have been identified to exert anticancer effects. Honokiol, a small molecule biphenolic compound purified from the medicinal herb *Magnolia officinalis* (Magnoliae Cortex), has been known to exert antithrombotic, antibacterial and anxiolytic effects (6–9). Studies have revealed that honokiol is able to inhibit tumor growth in animals and induce apoptosis in various types of cancer cells, including leukemia, hepatoma, prostate, lung and colon cells (10–13). More recently, honokiol has been shown to be capable of crossing the blood-brain barrier (BBB) and blood-cerebrospinal fluid barrier (BCSFB) and inhibiting brain tumor growth in the human U251 xenograft glioma model (14). However, little is known about the molecular mechanisms underlying the effects of honokiol against glioma cells.

The present study explored the effects of honokiol in DBTRG-05MG GBM cells to investigate whether autophagy is involved in the anticancer effects of the compound.

## Materials and methods

### Cell line and cell culture

The human GBM DBTRG-05MG cell line was maintained in RPMI-1640 medium (Gibco, Carlsbad, CA, USA) containing 10% fetal bovine serum, 0.01 M HEPES and 1 mM sodium pyruvate in a 37°C incubator with 5% CO_2_.

### Chemicals and reagents

Honokiol was dissolved in dimethyl sulfoxide (DMSO) at a concentration of 50 mM and stored at −20°C. Sulforhodamine B (SRB) was dissolved in phosphate buffer at a concentration of 5 mg/ml and stored at 4°C. SRB, DMSO and cadmium acetate were purchased from Sigma (St. Louis, MO, USA).

### Cell viability assay

The DBTRG-05MG cells were seeded at a density of 3×10^3^ cells/well in 96-well plates for 24 h. The cells were then treated with various concentrations of honokiol (6.25, 12.5, 25 and 50 μM) for 72 h. The cell numbers were determined using the SRB assay. Briefly, the cells were fixed in 10% trichloroacetic acid and stained with 0.4% SRB, a protein binding dye. Following incubation and washing with 1% acetic acid, the bound SRB was dissolved in 10 mM unbuffered Tris base and the optical density was measured at 562 nm using a microtiter plate reader.

### Analysis of sub-G_1_ apoptotic population and cell cycle distribution

One day after being seeded in a 6-well plate (1×10^5^ cells/ml, 2 ml/well), the cells were incubated with various concentrations of honokiol (12.5, 25 and 50 μM) for 72 h. The control groups were treated with phosphate-buffered saline (PBS) only. Upon harvesting, the cells were fixed in 70% ice-cold ethanol and stored at −20°C. The cells were then washed twice with ice-cold PBS and incubated with RNase and the DNA intercalating dye, propidium iodide (50 μg/ml). The percentages of the sub-G_1_ apoptotic population and cell cycle distribution were then analyzed using a flow cytometer (BD Biosciences, San Jose, CA, USA).

### Western blot analysis

The cells were seeded at density of 1×10^6^ cells/dish in a 10-cm dish for 24 h. To prepare the total cell extract, the cells were harvested at 72 h following the treatment described previously, then washed, lysed with lysis buffer [50 mM Tris-HCl (pH 7.4) 150 mM NaCl, 1% Triton X-100, 0.5% deoxycholate, 1 mM ethylenediaminetetraacetic acid (EDTA), 1 mM Na_3_VO_4_, 1 mM NaF and 2% cocktail] and cleared by centrifugation at 12,000 × g for 30 min at 4°C. Briefly, the cell pellets were lysed with protein extraction solution and incubated at −20°C for 20 min. Next, the cell lysates were centrifuged at 15,000 × g for 5 min and the total protein was collected. The protein concentration was measured using a protein assay kit (Strong Biotech, Taipei, Taiwan). Total protein (20 μg) was separated on a 10% SDS-PAGE gel and transferred to a PVDF membrane. Non-specific binding was blocked using 5% skimmed milk. Primary antibodies to detect RB (sc-102), poly(ADP-ribose) polymerase (PARP; sc-7150) and Bcl-x (S/L; sc-8392) were purchased from Santa Cruz Biotechnology, Inc. (Santa Cruz, CA, USA). Primary antibodies to phospho-RB (Ser807/811), Beclin-1 (#3738), LC3 (#4108) and glyceraldehyde 3-phosphate dehydrogenase (GAPDH; #2118) were purchased from Cell Signaling Technology (Beverly, MA, USA). The primary antibodies were detected using horseradish peroxidase (HRP)-conjugated anti-mouse, anti-rabbit or anti-goat secondary antibodies as appropriate, for 1 h at 25°C. The bound HRP-conjugated secondary antibodies were visualized using an Enhanced Chemiluminescence (ECL) Plus System (Millipore, Billerica, MA, USA).

## Results

### Honokiol inhibits the growth of GBM cells and induces apoptosis

**Figure 1:**
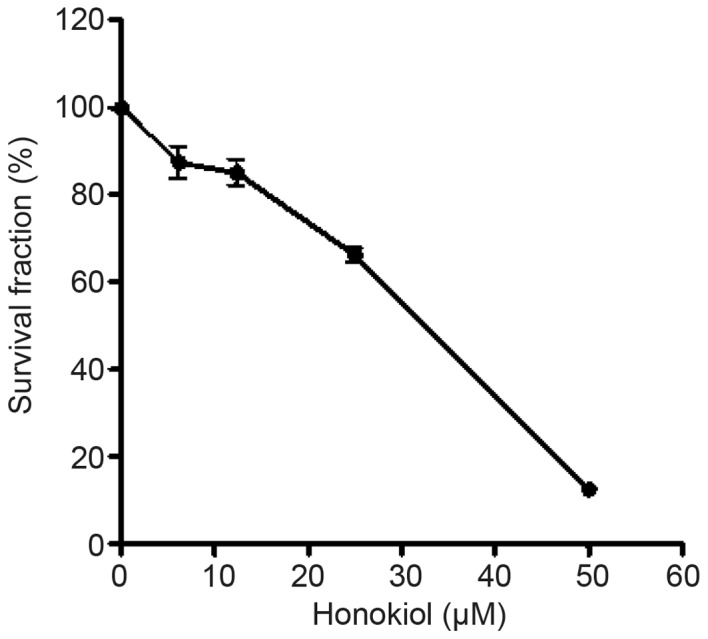
Effect of honokiol on DBTRG-05MG cell viability. The cells were incubated with various concentrations of honokiol for 72 h. The cell viability was determined by sulforhodamine B (SRB) assay.

**Figure 2:**
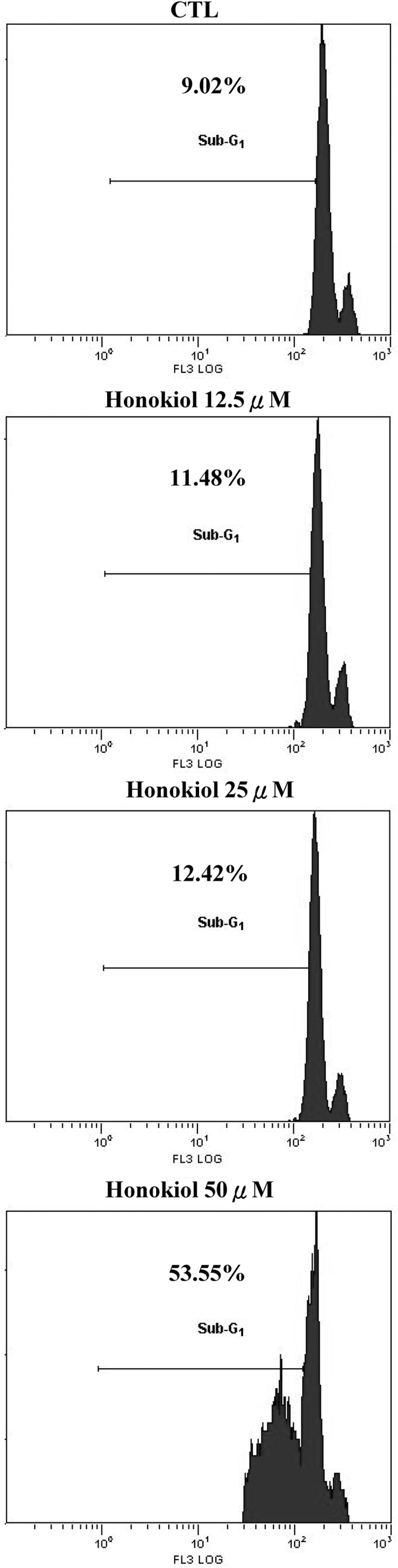
Honokiol-induced apoptosis in DBTRG-05MG cells. Following treatment with various concentrations of honokiol (0, 12.5, 25 and 50 μM) for 72 h, the cells were examined for apoptosis using propidium iodide (PI) staining and flow cytometry. The percentages of the apoptotic populations are shown in the histograms.

The SRB assay revealed that honokiol inhibited the growth of the DBTRG-05MG cells in a dose-dependent manner. Following 72 h of treatment, the IC_50_ of honokiol on the DBTRG-05MG cells was ~30 μM (Fig. 1). To investigate the apoptotic effects of honokiol on the GBM cells, the percentage of the apoptotic sub-G_1_ fraction was analyzed using a flow cytometer. Following the treatment with honokiol for 72 h, the percentage of the apoptotic sub-G_1_ fraction was 9.02, 11.48, 12.42 or 53.55% in the control and the cells treated with 12.5, 25 and 50 μM of honokiol, respectively (Fig. 2). A marked increase in the apoptotic fraction was not observed in the cells that were treated with ≤25 μM honokiol. When the dose increased to 50 μM, a massive sub-G_1_ fraction was observed, indicating that apoptosis only occurred at higher doses of honokiol (Fig. 2).

### Honokiol-induced apoptosis of GBM cells is associated with the downregulation of the Rb protein and cleavage of PARP and Bcl-x (S/L)

**Figure 3:**
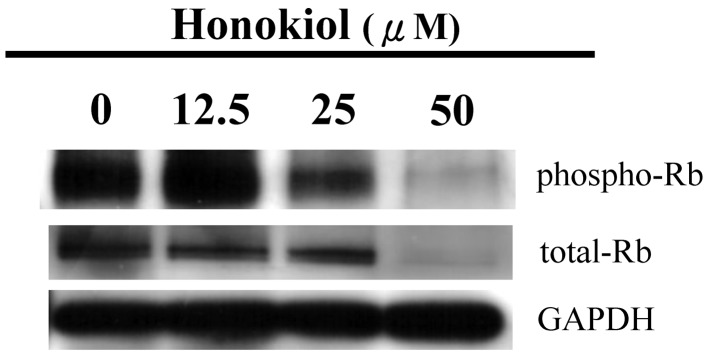
Effects of honokiol on Rb protein. The DBTRG-05MG cells were treated with honokiol at the indicated concentrations for 72 h and a western blot analysis was performed with antibodies that were specific for phospho- and total Rb protein. Glyceraldehyde 3-phosphate dehydrogenase (GAPDH) was used as internal control.

**Figure 4:**
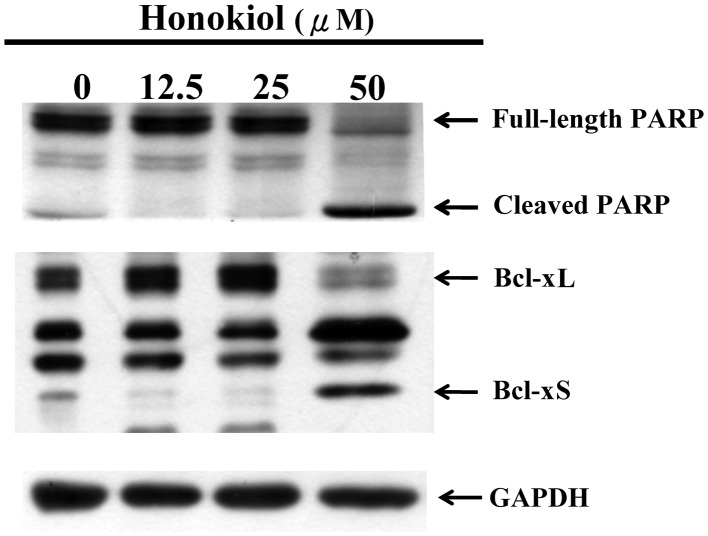
Effects of honokiol-induced cleavage in apoptosis-related proteins. The DBTRG-05MG cells were treated with honokiol at the indicated concentrations for 72 h and a western blot analysis was performed with antibodies that were specific for poly(ADP-ribose) polymerase (PARP) and Bcl-x (S/L). GAPDH, glyceraldehyde 3-phosphate dehydrogenase.

To improve our understanding of the events that are involved in honokiol-induced apoptosis, the effects of honokiol on the Rb, PARP and Bcl-x (S/L) proteins, which are known to regulate cell apoptotic cascades, were examined. At a dose of 25 μM, honokiol markedly decreased the phospho-Rb level, but did not significantly affect the total Rb level (Fig. 3). However, honokiol significantly decreased the phospho- and total Rb proteins when the dose reached 50 μM. This phenomenon was paralleled with the high level of apoptosis induced by the higher dose (50 μM) of honokiol shown in Fig. 2. Consistent with this, the cleavage of PARP, an apoptotic marker, and anti-apoptotic protein Bcl-x (S/L) were only observed in the cells that were treated with 50 μM honokiol (Fig. 4).

### Honokiol increases the level of autophagy markers in GBM cells

**Figure 5:**
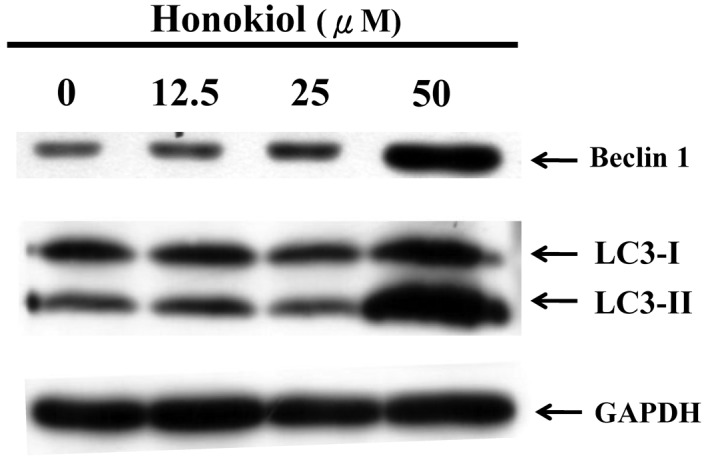
Honokiol-induced autophagy in DBTRG-05MG cells. The cells were treated with honokiol at the indicated concentrations for 72 h and a western blot analysis was performed with antibodies that were specific for Beclin-1 and LC3. GAPDH, glyceraldehyde 3-phosphate dehydrogenase.

To investigate if autophagy was involved in the honokiol-induced effects against GBM cells, the protein levels of two hallmarks of autophagy, namely Beclin-1 and MAP1LC3A (microtubule-associated protein 1A/1B light chain 3), were examined in the honokiol-treated cells. Honokiol markedly increased the levels of Beclin-1 and LC3-II at 50 μM (Fig. 5). Compared with the results that are shown in Figs. 2–4, as in the induction of apoptosis, autophagy was also markedly triggered by honokiol at a dose of 50 μM. The proautophagic effects of honokiol may play a crucial role in the anticancer effects against GBM cells.

## Discussion

To date, there have been no effective therapeutics for the treatment of GBM. Innovative strategies and agents must be investigated in order to conquer this life threatening disease.

Honokiol has been shown to exert antitumor effects in various types of cancer in animals (15,16). Furthermore, the toxicity of honokiol in normal peripheral blood mononuclear cells (PBMNCs) or primary cultured human cells is relatively low (17). As honokiol has been demonstrated to be able to cross the BBB and inhibit the growth of glioma in animal models, it may be regarded as a potential innovative therapy for treating GBM. However, little is known about the molecular mechanisms underlying the effects against glioma cells (14).

The present study explored the effects of honokiol in DBTRG-05MG GBM cells and demonstrated that type I (apoptosis) and II (autophagy) programmed cell death were involved in the effects against the cells. Consistent with other studies, the present data revealed that honokiol was able to induce apoptosis in the GBM cells, as reflected by the appearance of a massive apoptotic sub-G_1_ fraction. Hyperphosphorylation is able to inactivate the inhibitory effect of Rb on the E2F-1 transcription factor, leading to the progression of the cell cycle. The marked inhibition of phospho-Rb by honokiol at 25 μM may contribute to the significant decrease of GBM cell number at this dose without the significant induction of apoptosis. Chau and Wang (18) reported that the loss of phospho- and total Rb protein may sensitize cells to the induction of apoptosis. Similarly, in the present study, honokiol decreased the level of phospho- and total Rb proteins during the induction of apoptosis at a dose of 50 μM. In addition, the cleavage of PARP and anti-apoptotic protein Bcl-x (S/L) at this dose further confirmed this phenomenon. According to the literature, the cleavage of Bcl-x (S/L) is dependent on the activation of caspase-3-like protease (19,20). Thus, the cleaved Bcl-x (S/L) that was observed in the results of the present study indicated that caspase-3-dependent apoptosis may have occurred in the honokiol-treated GBM cells.

In addition to apoptosis, autophagy has been shown to be a potential target for cancer therapy. Proautophagic drugs are a promising class of compounds for counteracting tumor progression by favoring cancer cell death (21). A variety of chemicals, including temozolomide, the most effective agent for GBM treatment, have been reported to induce autophagy (22). In the present study, a marked honokiol-induced increase of the two hallmarks of autophagy, Beclin-1 and LC3-II, indicated the autophagy-inducing effects on the GBM cells. Depending on the magnitude of the induction of autophagy, honokiol may exert the effects of autophagic survival or cell death (23). Autophagy is a double-edged sword in tumorigenesis (24,25). As the marked autophagy of GBM cells was also triggered during the induction of apoptosis by honokiol at a dose of 50 μM in the present study, the modulation of autophagy by combining the compound with other agents may profoundly affect honokiol-induced cell death or apoptosis. Therefore, further investigation into a potential strategy combining the effects of autophagic modulating agents with honokiol in GBM cells is warranted.
